# Service Life and Early Age Durability Enhancement due to Combined Metakaolin and Nanosilica in Mortars for Marine Applications

**DOI:** 10.3390/ma13051169

**Published:** 2020-03-05

**Authors:** Ramiro García, Encarnación Reyes, Paula Villanueva, Miguel Ángel de la Rubia, Jaime Fernández, Amparo Moragues

**Affiliations:** 1 Department of Civil Engineering, Construction, School of Civil Engineering, Universidad Politécnica de Madrid, 28040 Madrid, Spain; garcia.ramiro@es.sika.com (R.G.); encarnacion.reyes@upm.es (E.R.); miguelangel.rubia@upm.es (M.Á.d.l.R.); jaime.fernandez.gomez@upm.es (J.F.); amparo.moragues@upm.es (A.M.); 2 Department of Mechanical Engineering, Chemistry and Industrial Design Department, E.T.S.I.D.I, Universidad Politécnica de Madrid, 28040 Madrid, Spain

**Keywords:** synergy nanosilica-metakaolin, durability at early ages, marine environment, service life, sustainability

## Abstract

The addition of a range of micro- and nano-particles to high-performance concrete has been the focus of recent research. At present, studies are mainly aimed at designing customised mortars, providing them with specific properties for each application. Improving the durability of mortars is one of the main objectives in such research, as a result of increasing environmental concern. The research presented herein analyses the synergistic effect of nanosilica and metakaolin as additives on the service life of cement-based mortars subject to aggressive environments (i.e., chloride exposure) at early ages. The effects of the additives on the durability properties of submerged samples after two and three days of curing were analysed. Tests were conducted on several different properties: resistivity, porosity, mechanical properties, chloride diffusion, and service life. It is observed that metakaolin and nanosilica exhibit a synergistic effect as additives, which is related to porosity refinement and chloride ion binding capacity, which contributes to enhanced resistance against chloride penetration from very early ages.

## 1. Introduction

The durability of structures has become an increasing concern, for both environmental and economic reasons [1,2,3]. In aggressive environments, such as locations with high chloride concentrations, it becomes particularly important to provide cementitious mixes with durable properties, which ensure an extensive service life. Lifetime prediction models take into consideration the resistance against penetration of aggressive agents, which is directly related to the porous network and, above all, to the pore interconnectivity. It is equally essential, in marine environments, to maximise the binding capacity of chloride ions, in order to restrain chloride penetration [4,5]. The chloride diffusion coefficient allows for prediction of the time until initiation of corrosion for a given chloride threshold and environment [6].

A number of studies in the literature have observed the valuable effect of pozzolanic additives, which limit the environmental impact of clinker manufacturing by means of reducing the amount of cement in the mixes [7]. These environmental benefits are increased when the pozzolanic materials are obtained from industrial waste materials, such as furnace slag or fly ash [8].

Metakaolin has been claimed to be a material with high pozzolanic performance. It is characterised by its fast strength evolution, accompanied by the densification of the microstructure at early ages [3,9,10,11,12]. In particular, the incorporation of metakaolin has been associated with an enhanced durability in marine environments, as a consequence of pore refinement together with the immobilization of chlorides by means of the formation of Friedel’s Salt [9,13]. Furthermore, improvements in terms of sustainability, thanks to the lower manufacturing temperatures compared to cement, have been highlighted [6,14].

Nanosilica has received increasing attention as an additive in mixes, due to its capacity for microstructure densification, refining porous networks by means of a filling effect, and contributing to the acceleration of the hydration process [15,16,17]. In addition, the capacity of nanosilica for chloride adsorption in calcium silicate hydrate (C–S–H) and aluminium-modified calcium silicate hydrate (C–A–S–H) gels is noteworthy [18,19,20].

There has been an important amount of research supporting the benefits of the use of micro- and nano-additives, in terms of the different properties of the resulting cement-based mortars and concrete. In the literature, the synergistic effect which has been found for a number of combinations of additives is especially relevant; this synergistic effect can contribute to further refinement of the porous network [9,21,22,23]. Combinations of micro- and nano-additives can also produce an increase in the hydrate gel content at early ages. Although it has been broadly acknowledged that metakaolin and nanosilica can improve the functional behaviour of cement blends, there are scarce references in the literature reporting the synergistic effect between these additives [24]. Furthermore, the lack of experimental studies on the durability of mortar samples including both metakaolin and nanosilica additions is noteworthy. There have been but a limited number of studies related to the durability behaviour of blends of cement with metakaolin alone [25] and with a combination of metakaolin and silica fume or combusted rice husk ash [26,27].

The present research aims to study a way to minimise chloride penetration at early ages by designing a mix with refined porosity and low pore interconnectivity while, at the same time, stimulating other processes that may result in improved durability properties. These processes are, mainly, maximisation of the physical and chemical binding capacity of chloride ions with the hydrate gel and facilitation of the formation of Friedel’s salt. Different mixes were designed with a combination of micro- and nano-additives in pursuit of this synergistic effect. Emphasis was put on durable performance at early ages, as it is generally considered that such ages are critical in off-shore construction. It should be noted that this type of construction may be subjected to an aggressive environment four or five days after the concrete is cast, or even at earlier ages. Our experimental results were employed to predict the service life for each mix, according to the Spanish standard for concrete EHE-08. Service life predictions were related with the values of electrical resistivity, in order to evaluate the relationship between the apparent diffusion coefficient and the results of the electrical resistivity tests.

## 2. Materials and Methods

An experimental study was conducted, in order to compare the mechanical and durability properties of cement-based mortars at early ages. All mixes employed cement type CEM III/A 42.5 N/SRC, in accordance with the standard EN 197-1:2011, which is generally selected for use in aggressive environments in the presence of chlorides. For each mix, both cylindrical and prismatic specimens were cast, according to UNE-EN-196-1, to allow for the testing of each property, according to its corresponding standard.

The control series was designed as a reference mortar, according to EN 197-1. It was used to obtain reference values, in order to better evaluate the enhancement obtained with the micro- and nano-additives. Different mixes of additives were tested, three of them with both nanosilica and metakaolin and one with only nanosilica. The cementitious components, including cement content and micro- and nano-additives, all had a total weight of 450 g. Table 1 collects the mix design parameters, indicating the percentage of cement weight substitution by each addition.

The use of nanosilica has been shown to improve the microstructure of C–S–H gels and aid in the creation of additional C–S–H gel. It can limit the growth of portlandite and monosulfoaluminate crystals, improving the aggregate–paste interface [28]. It is worth noting that the addition of nanosilica produces an increase in the water demand, due to the small size of the particles with a higher specific surface. To alleviate this problem, all mixes with additives also incorporated a superplasticizer that allowed for comparable workability in the different pastes. The superplasticiser Sika Viscocrete 20 HE (SIKA S.A.U., Madrid, Spain) was used, according to UNE-EN 934-2. This product is especially suitable for a high binder content and consists of an aqueous polycarboxylate solution with density 1.09 g/cm^3^. The amount recommended by the manufacturer for average workability is 0.2–0.8% of the cement weight, but this amount can be increased, up to 1–2% of the cement weight, with low water-to-cement ratios and for self-compacting concrete.

All mixes were produced with potable water from the urban supply of Madrid. The sand (Normensand, Beckum, Germany) was CEN-NOMRSAND for all cases, a product according to UNE-EN 196-1 with aggregate diameter ranging from 0.008–2 mm. A total of 1350 g of sand was added to each mix, which was comprised of 450 g of cement or cement plus additives.

Metakaolin (Metamax) was supplied by Engelhard, Iselin, NJ, USA. It has a SiO_2_ content equal to 51.52%, an Al_2_O_3_ content equal to 44.53% and has a very fine granulometry; its specific surface is 12.56 m^2^/g and its d_50_ is 3.24 μm. Three different nanosilica products were employed: Aerosil®200 (A200), Aerosil®OX50 (OX) and Sipernat®22S (SIP), all of them supplied by Evonik, North Rhine-Westphalia, Germany. Aerosil®200 is a pyrogenic, hydrophilic silica with a specific surface ranging from 200–225 m^2^/g and with d_50_ equal to 12 nm; it is broadly employed in coatings, unsaturated polyester resins, and laminating resins. Aerosil®OX50 is also a pyrogenic, hydrophilic silica, characterised by its small specific surface ranging from 35–65 m^2^/g; it has d_50_ equal to 40 nm, and has the qualities of reduced thickening and high purity. Sipernat®22S is a chemically-processed hydrophilic nanosilica, with a primary particle size between 5 and 100 nm, d_50_ equal to 14 μm, and specific surface of 180 m^2^/g; it possesses high absorption capacity, and is commonly used as a carrier and to reduce caking.

Specimen preparation consisted of manual mixing and homogenisation of the cement with metakaolin in hermetic plastic bags. Nanosilica was added to the water and to the superplasticizer. The additives were incorporated as partial substitution of the cement content in each case, as summarised in Table 1. The pastes were prepared in a pan mixer (Proeti C0086 Automatic Mixer, (Proeti S.A., Madrid, Spain), equipped with two speeds: 140 ± 5 or 275 ± 5 rpm for rotation and 62 ± 5 or 125 ± 5 rpm for planetary movement) for 30 s at a low rate. Then, the aggregated sand was added over 30 s, mixing at a low rate. This was followed by mixing at high rate for 30 s, a 90 s rest, and then mixing for 30 s at high rate again. Immediately after this, the mixes were cast into cylindrical and prismatic moulds for compaction with an Iberest Cib-801 (Ibertest, Madrid, Spain). Half of the mould was filled with the mix and 60 blows were applied, followed by complete mould filling and finishing with another 60 blows. Specimens were cured in a room with a controlled temperature of 22 ± 2 °C and a relative humidity of 99%; prismatic specimens were placed in the curing room immediately after casting, while cylindrical specimens were put in the curing room 24 h after unmolding.

**Figure 1:**
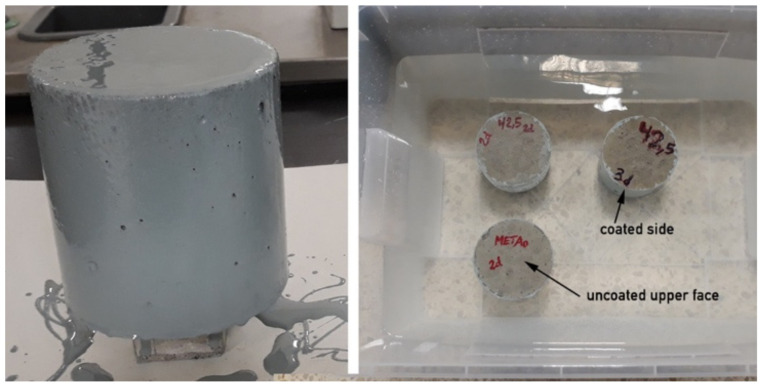
Sample preparation for chloride penetration tests.

Cylindrical specimens were employed for chloride penetration testing. The surface of each specimen was protected with an epoxy bi-component resin, Sikaguard 62 (SIKA S.A.U., Madrid, Spain), except for one of the circular sides, in order to guarantee that diffusion of Cl occurred only through the circular upper face of the cylinder. Figure 1 shows one sample after coating and the position of samples with the circular upper face of the cylinder without coating immersed in artificial seawater.

For the chloride penetration tests, a marine environment was simulated with artificial seawater according to Table 2, with a chloride concentration up to Cl^–^ 0.54 M.

## 3. Testing Procedures

Mechanical characterization and durability tests were performed, including resistivity, flexural strength, compressive strength, mercury porosimetry, chloride permeability, and chloride diffusion coefficient tests.

Electrical resistivity testing was performed on prismatic specimens according to UNE-83988-1, by generating an electric field passing through the specimen. An ARCON machine from GIATEC (Ottawa, ON, Canada) was used for these tests. Specimens were tested after 2, 3, 7, and 28 days in the curing room. Electrical resistivity of concrete is a material property that can be defined as the resistance against the flow of an electrical current [29]; therefore, it can be put in relation with the connected porosity, according to Ohm’s law. Connected porosity is associated with the resistance to movement of ions in the aqueous phase, as such movement only occurs through the connected concrete pores. Electrical resistivity values, thus, provide information about the porosity, which allows the penetration of aggressive agents (e.g., chlorides), especially at early ages, as has been reported in the literature [29,30,31].

The flexural strength was evaluated through a three-point bending test at the ages of 2, 7, and 28 days, at a loading rate between 40 and 60 N/s. The compressive strength was tested for each mix from the two halves of the specimens obtained from the flexural tests, at a rate of 50 ± 10 N/s. Mechanical characterization was done according to UNE-EN 196-1.

The microstructure and porosity of the different mixes were analysed through mercury intrusion porosimetry (MIP), according to ASTM D 4404. Data were collected from specimens at the ages of 2, 3, and 28 days in controlled temperature and humidity. Samples were extracted using a suitable core drilling machine (Ibarmia KL-25, Ibarmia Innovatek, S.L.U, Gipuzkoa, Spain). MIP was performed with a Micromeritics Autopore IV 9500 (Micromeritics Headquarters, Norcross, GA, USA), which has ability to measure pore diameters ranging from 0.006 to 175 μm with a controlled pressure of 228 MPa.

Chloride diffusion tests were performed, in order to evaluate both free chloride content (according to RILEM TC178-TMC) and total chloride content (according to UNE 14629). Using these data, it is possible to calculate the combined chloride as the difference between the total chlorides and free chlorides. After 28 days of exposure to the above-mentioned artificial seawater, ten slices were extracted from each specimen and collected as a powder; five of the samples at 1 mm depth and the other five samples at 2 mm depth from the top, using a suitable grinding wheel adapted to a drill machine (Bosch Professional CGS 28 LCE, Robert Bosch España S.L.U., Madrid, Spain) to allow for a polish thickness of 0.5 mm. Then, the samples were dried at a temperature of 105 ± 5 °C for 24 h, and finally put into a desiccator to prevent re-hydration of the powder until testing.

Free chloride content was obtained from dry samples with average weight of 1 g, located in 150 mL chemical vases. A stirrer and 10 mL of distilled water was added to each vase, and mixing was performed for 3 min. The sample was then filtrated into a flask with the assistance of filter paper (previously impregnated in 2 mL distilled water). After filtering, 2 mL of HNO_3_ conc. was incorporated, and the flask was filled up to 100 mL with distilled water. Once prepared, the samples were removed from the flask for analysis. Analysis was performed on samples under pH control with 0.5 mL of NaC_2_H_3_O_2_ and 20 mL of C_2_H_4_O_2_, using a Metrohm measurement tool which incorporates AgNO_3_ at known concentration and constant rate. The free chloride content could then be determined as a function of the amount of AgNO_3_ that was added.

Total chloride content was obtained from dry samples with average weight of 1 g, located in 250 mL chemical vases. The samples were mixed with a stirrer after the addition of 100 mm of distilled water and 5 mL HNO_3_ 1 M. The vases were covered with a watch glass and boiled for 3 min. The sample was analysed after cooling, following the same procedure as described for free chloride content, but also including NaC_2_H_3_O_2_ and C_2_H_4_O_2_.

## 4. Results and Discussion

This section collects and discusses the experimental results.

### 4.1. Electrical Resistivity

**Figure 2:**
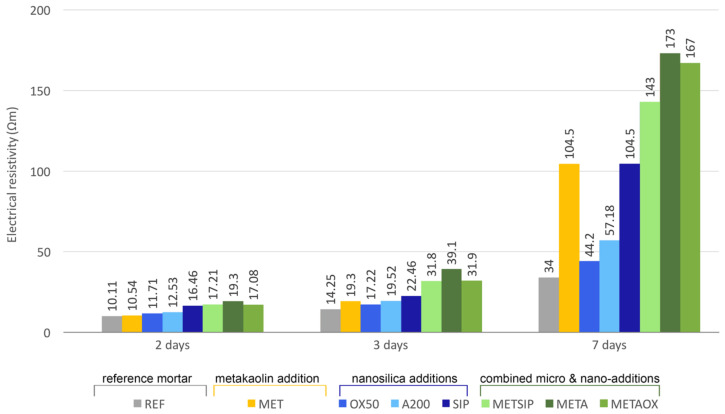
Evolution of electrical resistivity at early ages.

The values of electrical resistivity obtained from the experimental campaign are collected in Figure 2.

High values of electrical resistivity in mortars are related to enhanced durability, as they are an indicator of reduced porosity, preventing the penetration of aggressive agents. Our results are discussed in terms of enhancement with respect to the reference mortar, which presented the worst electrical resistivity performance with a low initial value and slow evolution (ranging from 10.11 Ωm at two days to 34 Ωm at seven days). The increase in electrical resistivity from 2 to 7 days was 236% in the reference mortar.

Electrical resistivity of the mix with only the addition of metakaolin (MET) had a considerable growth between three and seven days, with a total increase between two and seven days of 891%. Binary mixes with nanosilica (OX-50, A200 and SIP) had a slower evolution from two to seven days than mixes with metakaolin alone but, nevertheless, the resistivity at two days was higher than that of the reference mortar and MET mix. In terms of the binary nanosilica mixes, the best results were obtained for SIP, with a resistivity increase of 535% from two to seven days.

Ternary mixes combining micro- and nano-additives led to a faster evolution of electrical resistivity, as compared to binary mixes with nanosilica, ranging from 730.9% growth (METSIP) to 877.6% growth (METAOX) from day 2 to day 7. Together with the higher electrical resistivity of all ternary mixes than any of the binary mixes at two days, this resulted in the greatest performance at all ages of the META mix, followed by the METAOX and METSIP mixes and, finally, that of SIP. Remarkably, the electrical resistivity of the META mix at the age of three days was more than twice that of the reference mortar. Evolution was still positive with combined micro- and nano-additives after three days; the resistivities of both META and METAOX at seven days were more than four times that of the reference mortar, whereas the METSIP mix had an electrical resistivity at seven days that was three times that of the reference mortar. These results allow us to conclude that there is a positive effect of the combination of nanosilica and metakaolin on electrical resistivity at early ages.

Given the relatively limited enhancement of the electrical resistivity with only one additive, such as metakaolin or one nanosilica type alone, we decided not to include these mixes in the further studies of porosimetry, durability, and service life. Notwithstanding, mechanical characterisation was performed on all mixes, as the same specimens were employed in measuring the electrical resistivity (a non-destructive test) and the destructive tests in this phase of the research.

### 4.2. Mechanical Characterisation

The results of the compressive strength and flexural strength tests at different ages are summarised in Table 3.

As can be observed in Table 3, the studied additives scarcely affected the compressive strength at early ages. The results were slightly worse for the binary mix SIP with nanosilica and without metakaolin at two and seven days, while all ternary mortars with combined micro- and nano-additives performed better than the reference mortar at all ages. It should be noted that the values of compressive strength at seven days of the combined nanosilica–metakaolin mixes were higher than the compressive strength of the reference mortar at 28 days. Evolution of compressive strength was similar between seven and 28 days for all mixes. At the age of 28 days, the results of mixes with combined micro- and nano-additives were between 136% and 141% the compressive strength of the control series. The results for OX50 and METAOX exhibited a similar trend, indicating that nanosilica OX50 had a higher impact on mechanical properties (and, particularly, on compressive strength) than A200. The mechanical characteristics at 28 days of all mixes with additives, with respect to the reference mortar, are coherent with the action of nanosilica and metakaolin, given the pozzolanic activity of these additions, as reported in [3,9,10,11,12,15,16,17]. Additionally, the quick evolution of compressive and flexural strength with combined micro- and nano-additives seems to confirm a synergistic effect between them.

The evolution of the flexural strength was slower in the META mix, with values at two days, which were inferior to those of the reference mortar. All the other mixes had higher flexural strength at all ages, as compared to the reference mortar. At the age of 28 days, the mixes with additives presented values of flexural strength between 104% and 113% of the flexural strength of REF; the results of flexural strength were slightly higher for mixes with combined nanosilica and metakaolin.

### 4.3. Mercury Intrusion Porosimetry (MIP)

The evolution of porosimetry, as obtained from MIP tests, is collected in Table 4.

There were no significant differences between the different mixes in terms of total porosity. However, the total porosity of the reference mortar at two days was lower than that of all the mixes with additives. It should be noted that the analysis of the porosity does not depend only on the total porosity but also, and most importantly, on the pore size distribution. In addition, it is necessary to consider the logarithm differential intrusion, and to analyse it in relation to the pore size. These parameters were studied and compared for the reference mortar and for the mixes with combined micro- and nano-additives at early ages.

**Figure 3:**
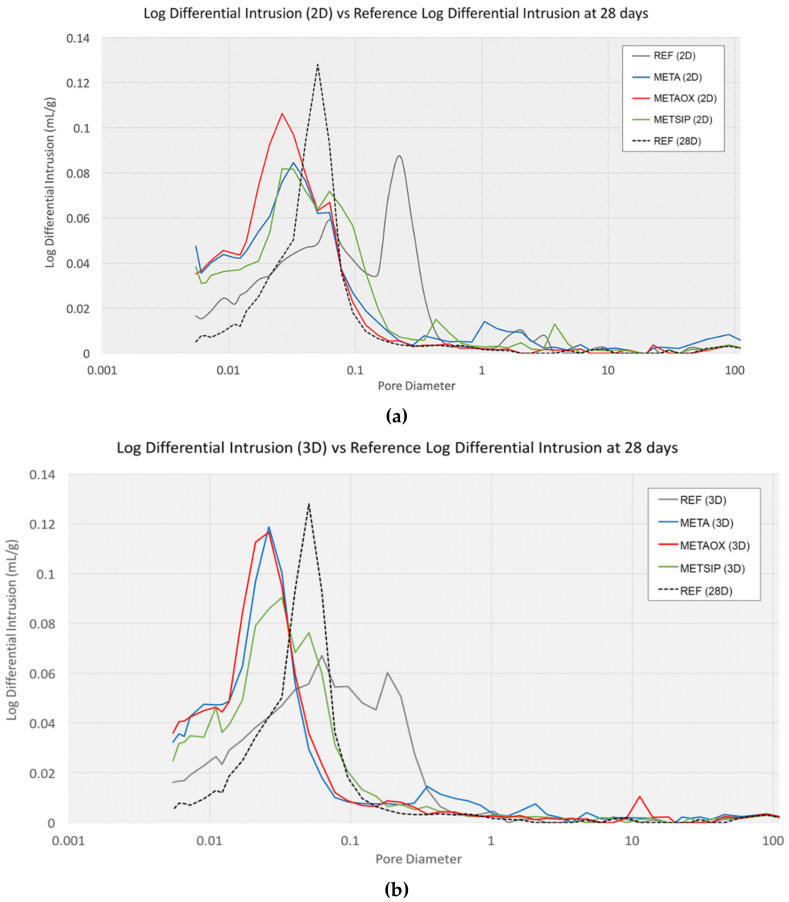
Logarithm differential intrusion vs. pore diameter at (**a**) two days and (**b**) three days, compared with the Logarithm differential intrusion vs. pore diameter of the reference mortar at 28 days.

The logarithm differential intrusion and intrusion volume, in relation with the pore size of the different mixes at the ages of two and three days, were compared with the results of the reference mortar at 28 days, as presented in Figure 3.

**Figure 4:**
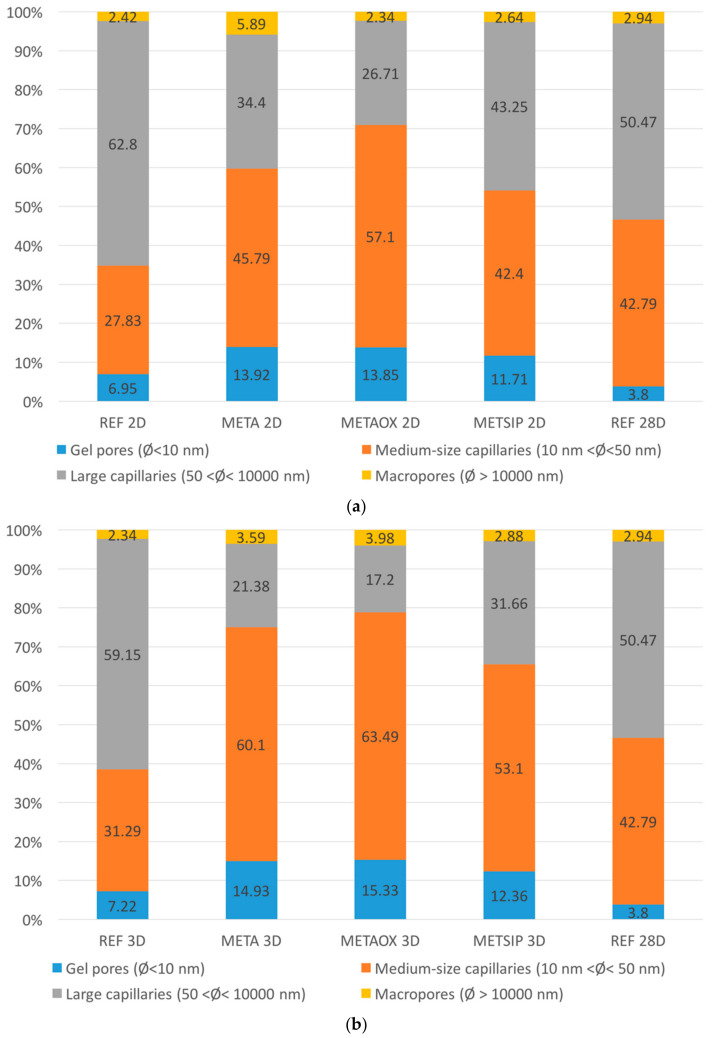
Pore size distribution of mixes with combined nanosilica and metakaolin at early ages and pore size distribution of reference mortar at 28 days. Distribution at (**a**) two days and (**b**) three days.

Together with these results, the pore size distributions at early ages of the studied mixes were analysed in order to compare them with the pore size distribution of the reference mortar at 28 days. These results are collected in Figure 4.

From the Figure 2 and Figure 3, it is possible to conclude that there was a refinement of the porous network in all studied combined micro- and nano-additives mixes. Pores were smaller at early ages in mixes with combined nanosilica and metakaolin than in the reference mortar at 28 days. In META and METAOX at early ages, it is possible to appreciate the change from a bimodal scheme to a predominant size, together with a refinement of the pore sizes. The behaviour of these two mixes at early ages is similar to that of the reference mortar at 28 days, but with even closer pores. It is possible to establish a relationship between this observation and the results from the electrical resistivity experiment discussed above.

The METAOX and META mixes presented very similar behaviour, in terms of pore size distribution; particularly after three days. The differences between these two mixes at two days can be explained by the different specific surface of the nanosilica that was employed in METAOX and META: the mix containing only A200 had a faster refinement of the pores than that containing OX-50, with a greater increase of mesopores from two to three days (and a corresponding diminution of macropores). Both METAOX and META had a refined porous network at early ages, with more than 60% of mesopores at the age of three days—a value which was higher than that attained by the reference mortar at 28 days. It is necessary to take into account that a high content of large capillaries and macropores is responsible for worse durability behaviour; thus, the results indicate that mixes with combined micro- and nano-additives may have better durability performance.

### 4.4. Chloride Penetration Testing

Chloride penetration data were collected at a range of depths for the different mixes with combined micro- and nano-additives and for the reference mortar. Specimens were subjected to an aggressive chloride environment after two and three days in controlled curing conditions, for comparison. Results at early ages of the different mixes were analysed in comparison with the reference mortar put into artificial seawater after 28 days in controlled curing conditions. The chloride diffusion coefficient was estimated according to ASTM C 1556-03.

**Figure 5:**
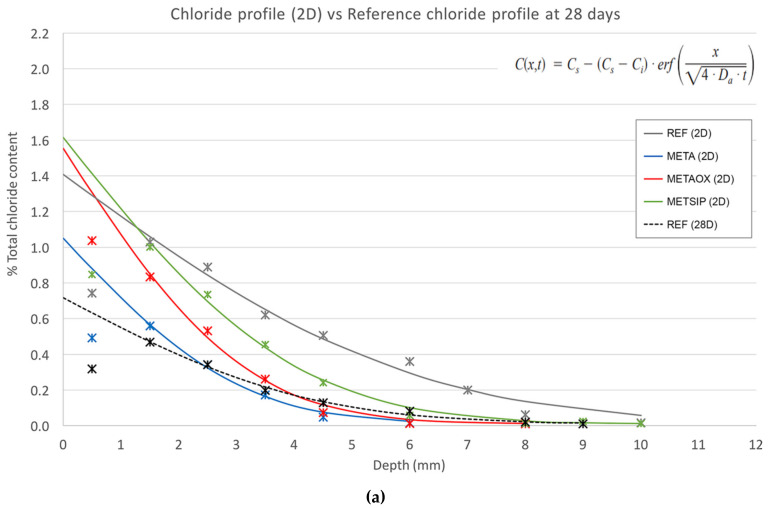

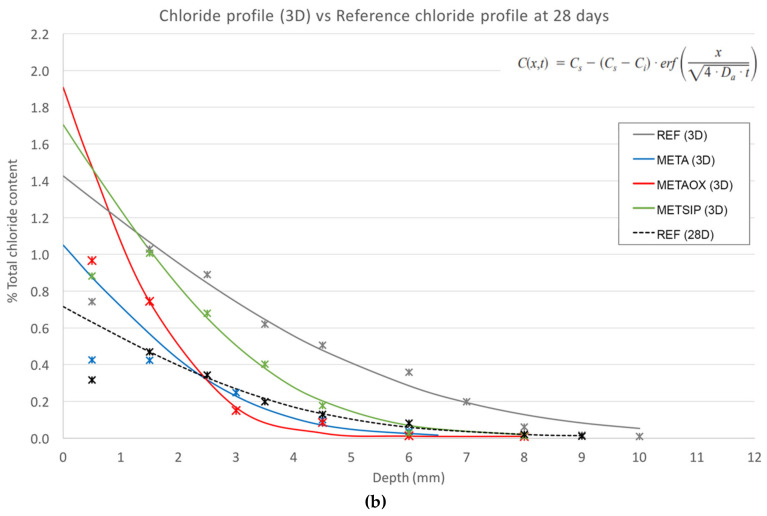
Chloride profiles at (**a**) two days and (**b**) three days for all samples and for reference mortar at 28 days.

Figure 5 shows the experimental results for the chloride profiles for the different mixes at early ages, in comparison with results of the reference mortar at 28 days.

The first layer was omitted in the regression analysis, according to the Nordtest method NT BUILD 443 for accelerated chloride penetration. The results of the apparent chloride diffusion coefficient at two and three days for the mixes with combined nanosilica and metakaolin and for the control series, together with the depth at which the limit of detection of chlorides by the equipment was reached, are collected in Table 5.

According to the results presented above, it can be concluded that the best mix, in terms of durability against chloride attack at early ages, was METAOX, as it had the lowest chloride diffusion coefficient at three days and considering its refined porous network which successfully prevents chloride penetration at greater depths. The low value obtained in this case was determined by the rapid reduction of the porosity of the material, which significantly hampers the advance of chlorides towards the interior. This motivates the increase in the surface concentration accompanied by lower content at greater depths. The high value of the surface concentration determines the low value of the coefficient.

The META mix demonstrated good durability behaviour, too, although its values are slowly lower than those of METAOX and there was no significant microstructural evolution between two and three days; this finding has been related to the fact that the nanosilica A200 is more kinetically reactive and, thus, leads to quicker porosity refinement.

**Figure 6:**
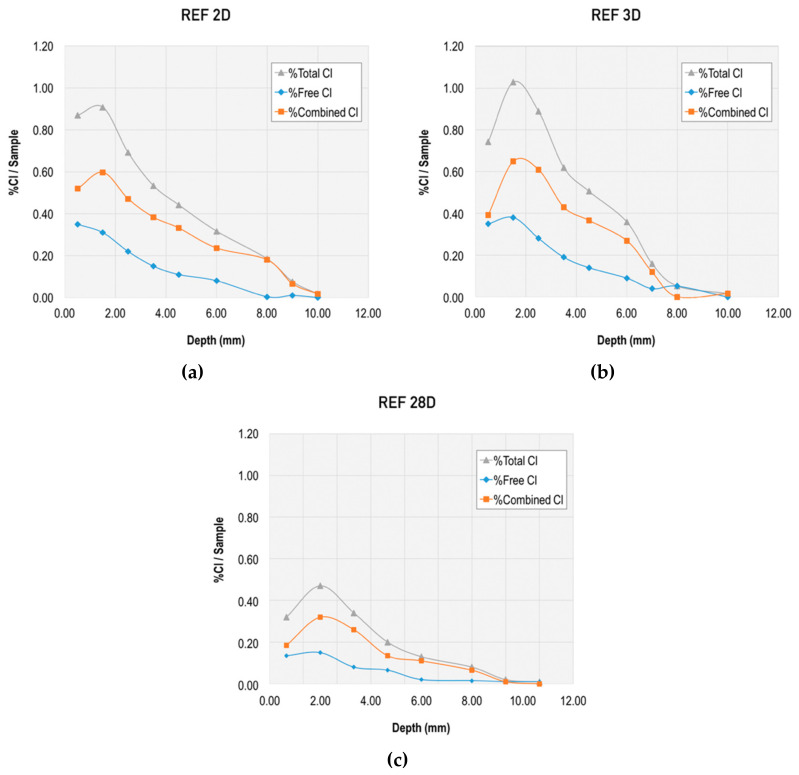
Total, free, and combined chloride content in reference mix at early ages: (**a**) at twodays, (**b**) at three days, and (**c**) at 28 days.

**Figure 7:**
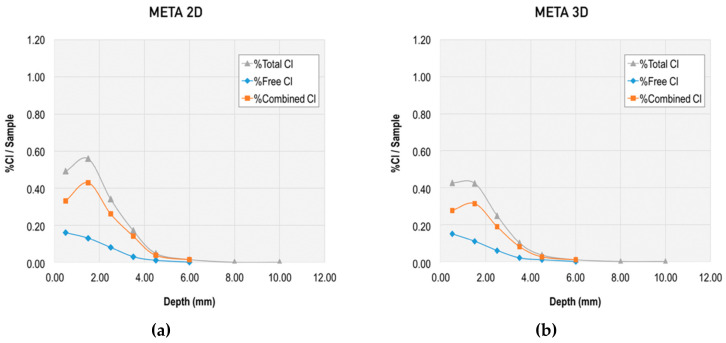
Total, free, and combined chloride content in META mix at early ages: (**a**) at two days and (**b**) at three days.

**Figure 8:**
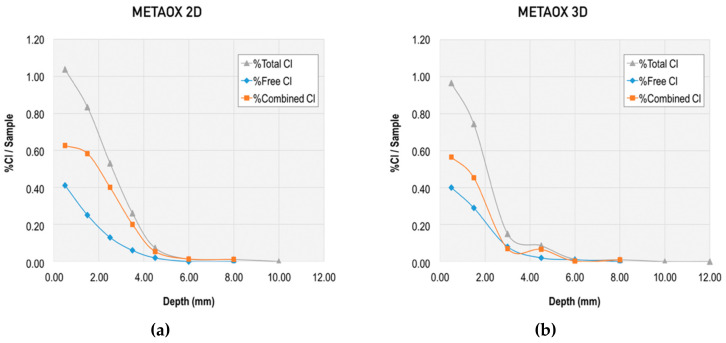
Total, free, and combined chloride content in METAOX mix at early ages: (**a**) at two days and (**b**) at three days.

**Figure 9:**
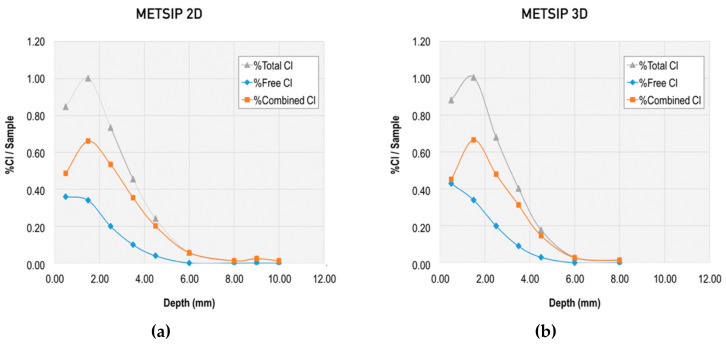
Total, free, and combined chloride content in METSIP mix at early ages: (**a**) at two days and (**b**) at three days.

Together with this analysis of the chloride diffusion coefficient, the binding capacity of chloride-ions for the different mixes was evaluated through the total chloride content and free chloride content, and the corresponding calculation of the combined chlorides. Results from this evaluation at different ages and at different depths are presented in Figure 6, Figure 7, Figure 8 and Figure 9.

**Figure 10:**
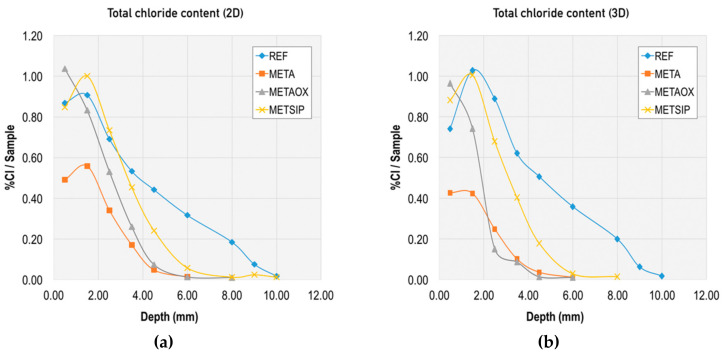
Total chloride content in different mixes at early ages: (**a**) at two days and (**b**) at three days.

**Figure 11:**
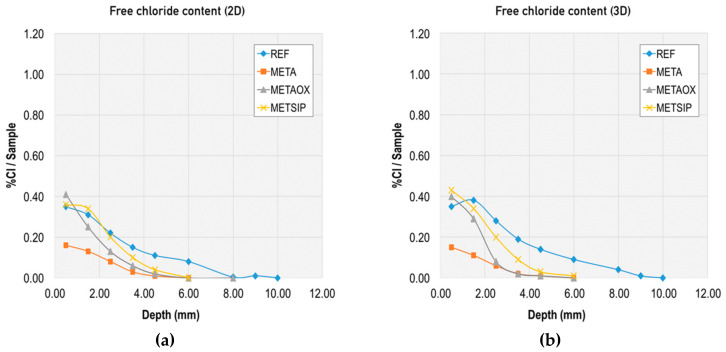
Free chloride content in different mixes at early ages: (**a**) at two days and (**b**) at three days.

For better comparison of the mixes, we also graphically collected the following: total chloride content at two and three days in the different mixes (Figure 10) and free chloride content at two and three days in the different mixes (Figure 11).

Figure 9 and Figure 10 allow for direct comparison of the penetration depth in the mixes. The reference mortar showed the largest penetration depth at both two and three days (10 mm). The minimum penetration depth was 6 mm at two and three days, which was obtained by the mixes META and METAOX.

The total chloride content was greater for the reference mortar, particularly at the 4 mm depth. The trend was similar for total and free chloride content, but the penetration depth was reduced in the free chlorides for mixes including nanosilica and metakaolin. Thus, the combination of micro- and nano-additives was able to limit chloride penetration and largely reduce the penetration depth, which was no greater than 6 mm for all mixes with combined additives at three days.

Within the studied mixes, the META mix had the lowest free chloride content, which suggests that it has a greater capacity of chloride binding. This chloride binding can be achieved either through chloride ion adsorption in the C–S–H gel or due to chemical binding forming Friedel’s salts; furthermore, the lower free chloride content in the META mix is related to its greater resistance against chloride penetration, thanks to the refined porosity at early ages (as presented in Figure 1 and Figure 2). It should be noted, however, that all of the studied combinations of nanosilica and metakaolin resulted in a reduced percentage of free chlorides, with respect to total chlorides at early ages.

There was a synergy between metakaolin and nanosilica that affected two different durability parameters: the refinement of porosity, preventing a massive chloride penetration from the initial ages of curing; and the chloride binding capacity, by combination of an ion adsorption mechanism and the promotion of the creation of Friedel’s salt. The capacity to combine chlorides in the presence of metakaolin and nanosilica was greater than the sum of the capacity of each of them independently, which indicates a certain synergy in the improvement of the durability behaviour in the simultaneous presence of both micro- and nano-scale functional additives. This aspect was further studied, as detailed below.

Archie’s law [32] defines the resistivity of a specimen as:

R_s_ = R_w_·a·Φ^−m^ (1)

where R_s_ is the resistivity of the mortar (mS/cm), R_w_ is the resistivity of the aqueous phase (mS/cm), Φ is the capillary porosity (% of volume) of the specimen, a is a parameter which depends on the rock type and is related to the path length of the current flow, and m is a parameter associated with the tortuosity of the material.

Using this equation, the m values were calculated for the studied mixes. The resistivity value of the aqueous phase has been obtained by the model proposed by NIST to calculate the conductivity of the aqueous phase, based on the composition of the cement and the dosage [33].

**Figure 12:**
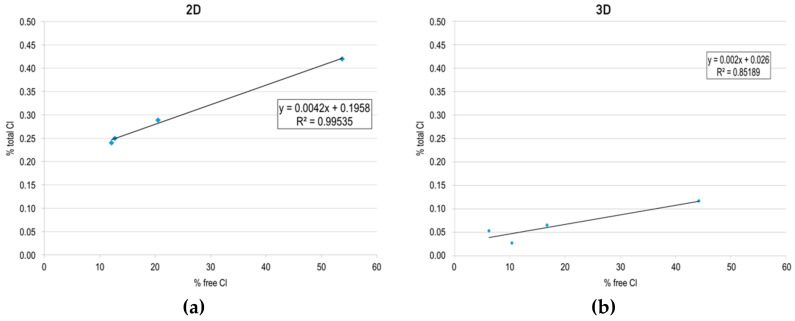
Calculated m value versus the apparent diffusion coefficient obtained for specimens submerged in an aggressive medium at early ages: (**a**) after two days and (**b**) three days of curing.

Figure 12 shows the results of the calculated m value versus the apparent diffusion coefficient obtained for the specimens submerged in the aggressive medium after two and three days of curing.

As can be observed from Figure 11a, the m value has a very good correlation with the apparent diffusion coefficient obtained (R^2^ = 0.995), which may indicate that, at this age (two days), the combination capacity does not have an important contribution; up to that age, the resistance against chloride penetration mainly depends on the tortuosity of the developing porous network.

In the case of the specimens submerged in the aggressive medium after three days of curing, the relationship between the apparent diffusion coefficient and m value shows a lower correlation (R^2^ = 0.899). This may indicate that, after three days, the combination capacity of the different mixtures begins to become significantly important, causing non-linearity in the relationship between the two parameters.

The results obtained might indicate that, in order to introduce the material into aggressive environments at very early ages, it is necessary to accelerate the process of refinement of the porous network. Until the age of three days of curing, the combining capacity of the cementitious matrix does not have a significant influence on the durability of the material.

## 5. Service Life Prediction

A number of international guides and regulations include procedures for the estimation of the service life of concrete structures and specify the design service life for different construction elements. These models are typically associated to one main aggressive agent in each environment. For this research, the Spanish code for structural concrete EHE-08 was employed for service life prediction, as this code includes a specific model for service life prediction in the presence of chlorides. The EHE-08 model distinguishes between an initiation period ( $t_{i}$ ), prior to the corrosion process, and a propagation period ( $t_{p}$ ), in which the reinforcement rebars are progressively corroded; the total service life is defined as the addition of the initiation period plus the propagation period [34].

The initiation period depends on a number of parameters, including the chloride penetration coefficient of the mix (*k*) and the cover depth of the reinforcement (*d*), for a given environmental condition. The relationship between the different components is expressed according to Equation (2):

(2) $$d=k\sqrt{t}$$

The chloride penetration coefficient can be calculated as follows:

(3) $$K_{Cl}=\alpha \cdot \sqrt{12\cdot D_{t}}\left(1-\sqrt{\frac{C_{th}-C_{b}}{C_{s}-C_{b}}}\right)$$

where $D_{\left(t\right)}$ is the effective chloride diffusion coefficient at a given age *t* (cm^2^/s); $C_{th}$ is the critical chloride content, in percentage of the cement weight; $C_{s}$ is the chloride concentration, in percentage of the cement weight, at the concrete surface; and $C_{b}$ is the chloride content in the raw materials of the mix. $D_{\left(t\right)}$ can be calculated, according to Equation (4):

(4) $$D_{t}=D_{t0}\cdot {\left(\frac{t_{0}}{t}\right)}^{n}$$

where $D_{t0}$ is the diffusion coefficient at a given age $t_{0}$ and n is an age factor, which can be considered to be equal to 0.5 unless specifically tested. It should be noted that $D_{t0}$ can be collected from experimental testing or taken from the reference values in EHE-08 for each cement type and water-to-cement ratio.

For the estimation, a value of $C_{th}$ equal to 0.6% was adopted (reference value for reinforcing rebars in EHE-08 [34]). Similarly, $C_{s}$ has been defined, in the code, for each environmental condition; in the considered case, it was equal to 0.72.

The initiation period for the different mixes was calculated for the experimental apparent and effective chloride diffusion coefficients at a penetration depth equal to 20 mm, as reported in Table 6, according to Equation (5) from EHE-08 [34]:

(5) $$t={\left(\frac{d}{K_{Cl}}\right)}^{2}$$

where t is the corrosion initiation time (in years), *d* is the concrete cover thickness (mm), and *K_Cl_* is the apparent or effective chloride penetration factor (m/year).

The propagation period is the time elapsed between the initiation of the corrosion and the moment when considerable section loss occurs. It can be calculated as a function of the cover depth d , rebar diameter ∅ , and the corrosion speed rate in μm/year. In the Spanish standard EHE-08, values for the speed corrosion rate for a range of environmental exposures have been determined; for the studied case (IIIb type environment), the corrosion speed rate is 4 μm/year. The propagation period can be calculated using Equation (6):

(6) $$t_{p}=\frac{80}{\emptyset }\cdot \frac{d}{v_{corr}}.$$

It should be noted that the propagation period does not depend on the mix properties and, so, it is the same for all studied mortars. Given a reinforcement cover of 40 mm (which is the minimum value for the IIIb type environment, according to the EHE-08), and for rebars with diameter equal to 16 mm, the propagation period would be 50 years.

**Figure 13:**
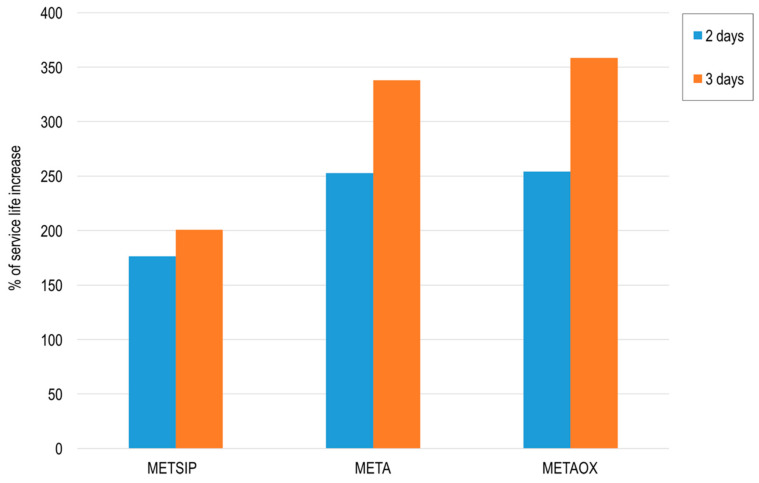
Percent increase of the initiation period of the studied mixes, with respect to the reference mortar, for ages of two and three days (calculated from the effective diffusion coefficient).

As mentioned above, the propagation period cannot be altered by changing the durability properties of the mix. In this sense, the change in the initiation period matters more, as it can lead to better use of the material, allowing smaller cover depths. The initiation periods were much longer for the mixes with combined micro- and nano-additives than that of the reference mortar. This increase is graphically presented, in Figure 13, for early exposure to the marine environment (two and three days).

It can be observed that all mixes largely fulfil the requirement of a 50 year service life, regardless of the 50 years of the propagation period.

**Figure 14:**
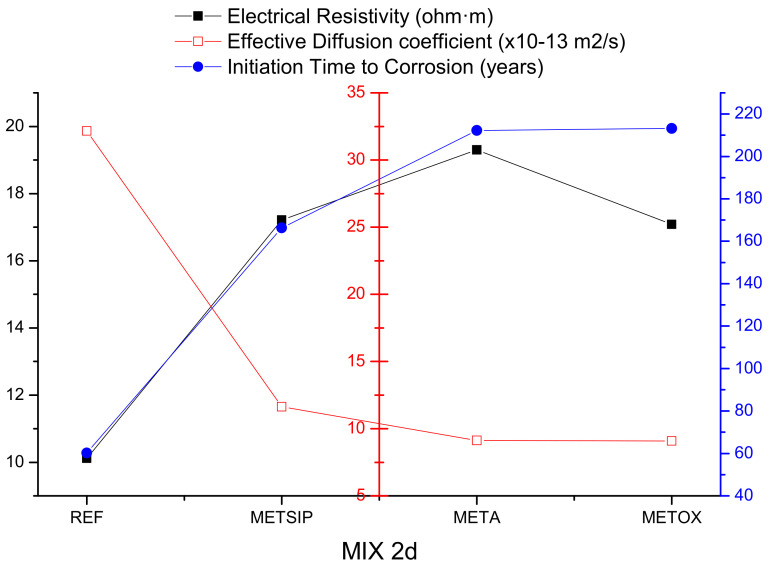
Electrical resistivity, effective diffusion coefficient, and corrosion initiation time for mixes with two days of curing time.

**Figure 15:**
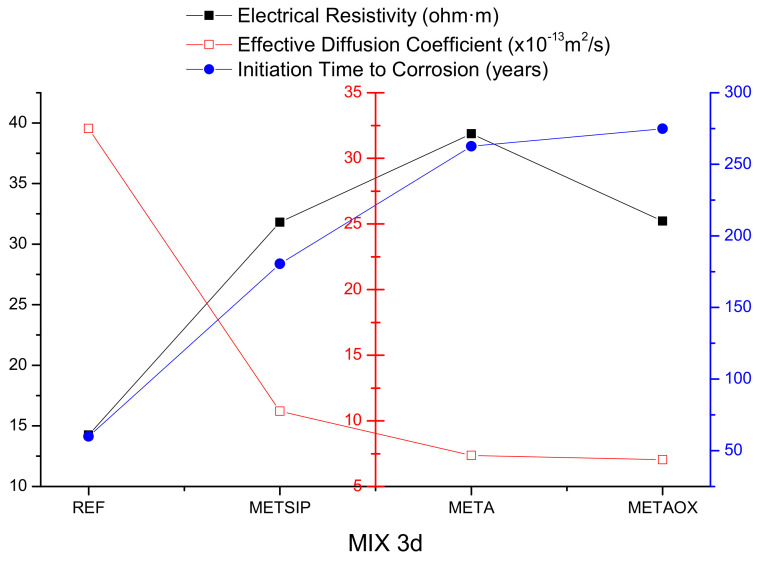
Electrical resistivity, effective diffusion coefficient, and corrosion initiation time for mixes with three days of curing time.

The electrical resistivity experimental results were put into relation with the effective diffusion coefficient used for service life predictions in the aggressive environment with chlorides, in order to estimate the initiation time for corrosion, according to the EHE-08. Given that the motivation for this research was the use of mixes that could be put into an aggressive environment at an early age, the comparison was carried out for two and three days of curing. The results of this comparison are collected in Figure 14 and Figure 15.

From Figure 13 and Figure 14, it can be observed that high values of electrical resistivity resulted in low values of effective diffusion of chlorides. There was no direct proportionality between the two properties, as there were several parameters affecting the resistance against penetration of chlorides; however, it became clear that both refined porous networks and binding capacity worked together to limit the chloride penetration and thus propitiate a delay in the corrosion initiation time.

## 6. Conclusions

An experimental campaign analysing the effects of combined micro- and nano-additives (metakaolin and nanosilica) on the durability properties of mortars exposed to marine environments at early ages has been presented in this paper. The results have been put in relation to the potential enhancement of service life of the resultant materials. The following conclusions can be drawn:

- Electrical resistivity testing is a non-destructive test which allowed us to select the most promising mixtures for a better functional response. From our experimental research, it was confirmed that mixtures with high electrical resistivity were those that showed the best durability behaviour under exposure to an aggressive medium (e.g., with high chloride content).
- The combined addition of nanosilica and metakaolin resulted in a refined porous network in all the analysed samples, with high compressive strength. There was a diminution of the pore size in the studied mixes, with respect to the reference mortar. The META and METAOX mixes presented the most refined porous networks at two and three days, with great similarities between the two mixes at three days. This trend continued with the hydration process, as observed at 28 days.
- Ternary mixtures with combined micro- and nano-additives had lower chloride diffusion coefficients than the reference mortar at early ages. The slower acquisition of resistance against chloride penetration by the reference mortar made it worse than the studied mixes, even after 28 days.
- The synergistic effect that occurs when two functional particles, such as metakaolin and nanosilica, are added to cement-based mortars in combination can lead to a considerable increase in the service life, due to the refinement of porosity at early curing ages and to an increase in the chloride binding capacity.
- META and METAOX mixes presented the highest electrical resistivities at early curing ages, as well as the lowest chloride diffusion coefficients, obtaining a remarkable extension in service life when considering early environmental exposure.
- The results of the experimental research presented herein allow us to conclude that ternary cementitious mixes utilising combined metakaolin and nanosilica are suitable for aggressive environmental exposure at very early ages, due to their hydration process and early age properties, which make them very useful for floating port drawers, off-shore platforms, and other marine construction works.

## Figures and Tables

**Table 1:** Mix design parameters.

Mix	Cement Content	Nanosilica (Sipernat 22S)	Nanosilica (Aerosil A200)	Nanosilica (Aerosil OX50)	Metakaolin	W/C	Superplasticiser
REF	100%	N/A	N/A	N/A	N/A	0.5	N/A
SIP	98%	2%	N/A	N/A	N/A	0.5	1.22 g
METSIP	90%	2%	N/A	N/A	8%	0.5	2.43 g
METAOX	90%	N/A	1.5%	0.5%	8%	0.5	2.43 g
META	90%	N/A	2%	N/A	8%	0.5	2.43 g
MET	92%				(8%)	0.5	1.22g
A200	98%		(2%)			0.5	1.22g
0X50	98%			(2%)		0.5	1.22g

**Table 2:** Chemical composition (molarity) of artificial seawater (left) and natural seawater (right).

Chemical Compound	Molarity	Chemical Compound	Molarity
NaCl	0.410 M	Cl^−^	0.547 M
MgCl_2_	0.105 M	Na^+^	0.469 M
Na_2_SO_4_	0.028 M	Mg^2+^	0.053 M
CaCl_2_	0.020 M	S^2–^	0.028 M
KCl	0.008 M	Ca^2+^	0.010 M
NaHCO_3_	0.0026 M	K^+^	0.010 M

**Table 3:** Mechanical properties at 2, 7, and 28 days.

Mix	Compressive Strength (MPa)			Flexural Strength (MPa)		
Age	2 days	7 days	28 days	2 days	7 days	28 days
REF	26	39	43	47	69	75
SIP	24	38	46	51	71	78
METSIP	28	51	61	51	75	80
META	26	46	59	41	70	83
METAOX	27	50	60	53	76	85
MET	28	51	63	55	83	92
A200	26	44,5	55	52	75	92
0X50	26	47.5	61.5	51	73.5	94

**Table 4:** Results from mercury intrusion porosimetry at different ages.

Mix	2 Days (%)	3 Days (%)	Diminution (%)	28 Days (%)	Diminution (%)
REF	17.53	16.87	3.76	13.18	24.81
META	18.17	16.94	6.77	-	-
METAOX	18.04	16.87	6.48	-	-
METSIP	18.25	16.84	7.73	-	-

**Table 5:** Chloride diffusion coefficient at two and three days.

Mix	2 Days		3 Days	
	Apparent Chloride Diffusion Coefficient (E-13 m^2^/s)	Depth (mm)	Apparent Chloride Diffusion Coefficient (E-13 m^2^/s)	Depth (mm)
REF	53.728	11	44.211	10
META	11.992	6.5	10.352	6.5
METAOX	12.625	6.5	6.173	6
METSIP	20.508	10	16.700	8

**Table 6:** Initiation period for the different mixes.

Sample	Apparent Diffusion Coefficient *D_a_* (×10^−13^ m^2^/s)	*t_i_* (Years) at a Penetration Depth of 20 mm, Calculated from *D_a_*	Effective Diffusion Coefficient *D_e_* (×10^−13^ m^2^/s)	*t_i_* (Years) at a Penetration Depth of 20 mm, Calculated from *D_e_*
Ref 2d	57.753	33.51	32.158	60.18
Ref 3d	54.938	35.23	32.273	59.97
Ref 28d	22.854	84.55	13.006	148.81
METSIP 2d	20.318	95.26	11.638	166.30
METSIP 3d	16.622	116.44	10.724	180.48
META 2d	11.992	161.39	9.120	212.22
META 3d	10.352	186.96	7.371	262.58
METAOX 2d	12.625	153.30	9.078	213.22
METAOX 3d	6.173	313.53	7.041	274.88
