# Correction: García et al. Service Life and Early Age Durability Enhancement due to Combined Metakaolin and Nanosilica in Mortars for Marine Applications. *Materials* 2020, *13*, 1169

**DOI:** 10.3390/ma15155144

**Published:** 2022-07-25

**Authors:** Ramiro García, Encarnación Reyes, Paula Villanueva, Miguel Ángel de la Rubia, Jaime Fernández, Amparo Moragues

**Affiliations:** 1 Department of Civil Engineering, Construction, School of Civil Engineering, Universidad Politécnica de Madrid, 28040 Madrid, Spain; garcia.ramiro@es.sika.com (R.G.); encarnacion.reyes@upm.es (E.R.); miguelangel.rubia@upm.es (M.Á.d.l.R.); jaime.fernandez.gomez@upm.es (J.F.); amparo.moragues@upm.es (A.M.); 2 Department of Mechanical Engineering, Chemistry and Industrial Design Department, E.T.S.I.D.I, Universidad Politécnica de Madrid, 28040 Madrid, Spain

The authors would like to make a correction in a recently published paper [1].

## Removal Affiliation(s)

In the published publication, there was an error regarding the affiliation(s) for “Ramiro García”. In addition to affiliation(s) “1”, should be “Department of Civil Engineering, Construction, School of Civil Engineering, Universidad Politécnica de Madrid, 28040 Madrid, Spain; garcia.ramiro@es.sika.com”. The authors apologize for any inconvenience caused and state that the scientific conclusions are unaffected. This correction was approved by the Academic Editor. The original publication has also been updated.
